# Assessment of the retinal vasculature in healthy Chinese preschool children aged 4–6 years old using optical coherence tomography angiography

**DOI:** 10.1186/s12886-021-02154-4

**Published:** 2021-12-02

**Authors:** Lu Xiang, Yingming Zhou, Yanwei Chen, Siyu Jiang, Chunli Fei, Yangyang Wang, Yang Bai, Xuan Zhang, Kai Li, Xi Shen

**Affiliations:** 1 Department of Ophthalmology, Ruijin Hospital, Shanghai Jiaotong University of Medicine, Shanghai, China; 2 Department of Ophthalmology, Wuxi Branch of Ruijin Hospital, Shanghai Jiaotong University of Medicine, Shanghai, China

**Keywords:** Healthy preschool children_1_, Optical coherence tomography angiography_2_, Vessel density_3_, Macula_4_, Optic disc_5_

## Abstract

**Purpose:**

To establish normal parameters of macular and optic disc vasculature by optical coherence tomography angiography (OCTA) in healthy preschool children aged 4–6 years old in China. OCTA reflects retinal metabolism and development in children at these ages and could be used clinically and in future studies to aid diagnosis and prediction of retinal abnormalities and developmental stagnation.

**Methods:**

In this cross-sectional study, we measured foveal, parafoveal, and perifoveal vessel density in the superficial capillary plexus (SCP); the deep capillary plexus (DCP), the foveal avascular zone (FAZ), and the radial capillary peripapillary (RPC) in the optic disc using investigational spectral-domain OCTA. The magnification effect of the FAZ area and microvasculature measurements was corrected by Littman and the modified Bennett formula.

**Results:**

A total of 242 eyes (116 males and 126 females, 5.31 ± 0.73 years) were recruited for the analysis. The mean macular vessel density was 48.10 ± 2.92% and 48.74 ± 6.51% in the SCP and the DCP, respectively. The RPC vessel density was 47.17 ± 2.52%, 47.99 ± 4.48%, and 48.41 ± 3.07% in the whole image, inside disc, and peripapillary, respectively; and the mean FAZ area was 0.28 ± 0.11 mm^2^. A significant difference between male and female participants was found in the retinal vasculature (DCP, SCP, and RPC). None of these parameters were significantly different in age (*P* > 0.05), except that DCP slightly increased with aging. The right and left eyes had good consistency in the parameters of the macula and optic disc.

**Conclusions:**

Our study establishes the macular and optic disc OCTA reference values in 4- to 6-year-old healthy preschool children. They may be used in longitudinal OCTA studies and clinical applications.

## Introduction

The retinal vascular system supports a majority of metabolic requirements, including transporting oxygen and nourishing the retina [1]. Normal visual function relies on an intact retinal vascular system. Quantitative studies have revealed that fundamental changes in the microvasculature occur in the early stage of retinal [2] and optic disc diseases [3].

Over the past 50 years, fundus fluorescein angiography (FFA) and indocyanine green angiography (ICGA) have been regarded as the gold standard in diagnosing retinal [4] and choroidal diseases [5]. However, the dyeable and invasive characteristics of these procedures place limitations on frequent monitoring and repeated quantification of the vasculature, especially in children. OCT angiography (OCTA) has the inherent nature of high resolution and noninvasiveness [6, 7] and allows for precise and reproducible detection in both the macula [8–10] and optic disc [11, 12]. Qualitative and quantitative studies have been related to vasculature in the normal eyes [13], various retinopathies [14–16], and glaucoma [13, 17, 18] of healthy adults. Relevant factors, such as age and sex, were fully researched in these adult studies. However, explorations on the characteristics of the macular and optic disc vascular systems in healthy children are sparse. Some studies have revealed that some congenital and pediatric retinal diseases (such as familial exudative vitreoretinopathy (FEVR) [19, 20], retinopathy of prematurity (ROP) [21], and Coats’ disease [22]) induce changes in structure and vasculature even in an asymptomatic stage. The preschool stage is a critical period for retinal and refractive development and plays a vital role in forming normal visual function [23]. Structural changes may not be recognized by clinical examination in some retinal diseases and glaucoma, but vascular changes brought to bear by OCTA could reveal the pathophysiology. Thus, it is crucial to obtain baseline OCTA data and explore the factors affecting OCTA parameters in healthy children. To date, we were unaware of previous research focusing on retinal vasculature in normal eyes of 4–6 years old children worldwide. Although some studies contained these ages [24, 25], we owned a larger specimen size of this period. What’s more, we corrected the magnification effect of the OCTA parameters to ensure the accuracy of our results.

Thus, our research has two aims. The first is to provide reference values ​​of the retinal vasculature of healthy children aged 4–6 years old in China. Our research will also investigate relevant factors (such as gender, age and anterior segmental parameters) to supplement the existing studies. The findings of the current study could be helpful for clinical application by detecting deviations in the retinal vasculature.

## Materials and Methods

This cross-sectional study was carried out in the Wuxi Branch of Ruijin Hospital, Shanghai Jiaotong University School of Medicine. It was approved by the institutional ethics committee of Ruijin Hospital and adhered to the tenets of the Declaration of Helsinki. We obtained written informed consent from the parents or guardians before the study examination.

A total of 242 healthy Chinese subjects aged 4–6 years old were enrolled during June through November 2020 and examined by experienced optometrists. Participants completed comprehensive ocular examinations including determination of best-corrected visual acuity, slit-lamp biomicroscopy, autorefraction (ARK-1a/ARK-1, Nidek, Tokyo, Japan), and non-contact intraocular pressure measurement (TX-20P, Canon, Tokyo, Japan). Axial length, anterior chamber depth, central corneal thickness, anterior chamber depth and crystalline lens thickness were obtained with optical biometry scans (Lenstar LS900, Haag-Streit AG, Koeniz, Switzerland). Atropine was instilled in both eyes three times a day for three consecutive days before data collection to facilitate cycloplegic refraction and fundoscopy. Inclusion criteria included the following: healthy subjects aged 4–6 years old; no major systemic diseases or eye diseases; a BCVA of 6/12 or better; spherical equivalent between + 1.0 and + 2.5D in 4-year-old child, between + 0.5 and + 2.0D in 5- and 6-year-old child, astigmatism less than 1.0 D; intraocular pressure less than 21 mmHg. Exclusion criteria included children with pathological anisometropia (spherical difference more than 1.50D), eye surgery history, amblyopia, nystagmus, retinal disease, inflammation, prematurity, neurologic disease, or systemic conditions in the ophthalmic examinations.

OCTA images were captured by RTVue Avanti spectral-domain optical coherence tomography equipped with AngioVue software (Version 2017,1,0,155; Optovue, Inc., Fremont, CA, USA) in the high-definition (HD) disc scan (4.5 × 4.5mm) and HD retina scan (6.0 × 6.0mm) modes. The images of the eye with better scan quality was chosen for further study, or if equivalent, then the eye was chosen randomly. Description of the OCTA measurements and segmentation of the retinal and vascular layers was reported in previous studies [26–30]. The magnification effect of the structural and vascular parameters in the retina was corrected using Littman and the modified Bennett formula [31]. Acquired images were eliminated when the scan quality was less than six.

We completed the statistical analysis using SPSS (SPSS for Mac, version 22.0; IBM SPSS, Inc., Chicago, IL, USA). Descriptive statistics of parameters were calculated by means, standard deviations (SD), ranges, and percentages. A one-sample Kolmogorov-Smirnov test was applied before the significance test, and all continuous variables showed normal distributions. The independent sample t-test was used to assess gender or eye-type differences, and one-way ANOVA was used to distinguish the differences between ages. Univariate and multivariate linear regression analyses were performed to determine the correlation between vessel density and anterior segment parameters. Simultaneously, the Spearman correlation coefficient was used to analyze the correlation between retinal thickness and vessel density in both the macula and optic disc. A *P*-value less than 0.05 was considered statistically significant.

## Results

**Fig. 1:**
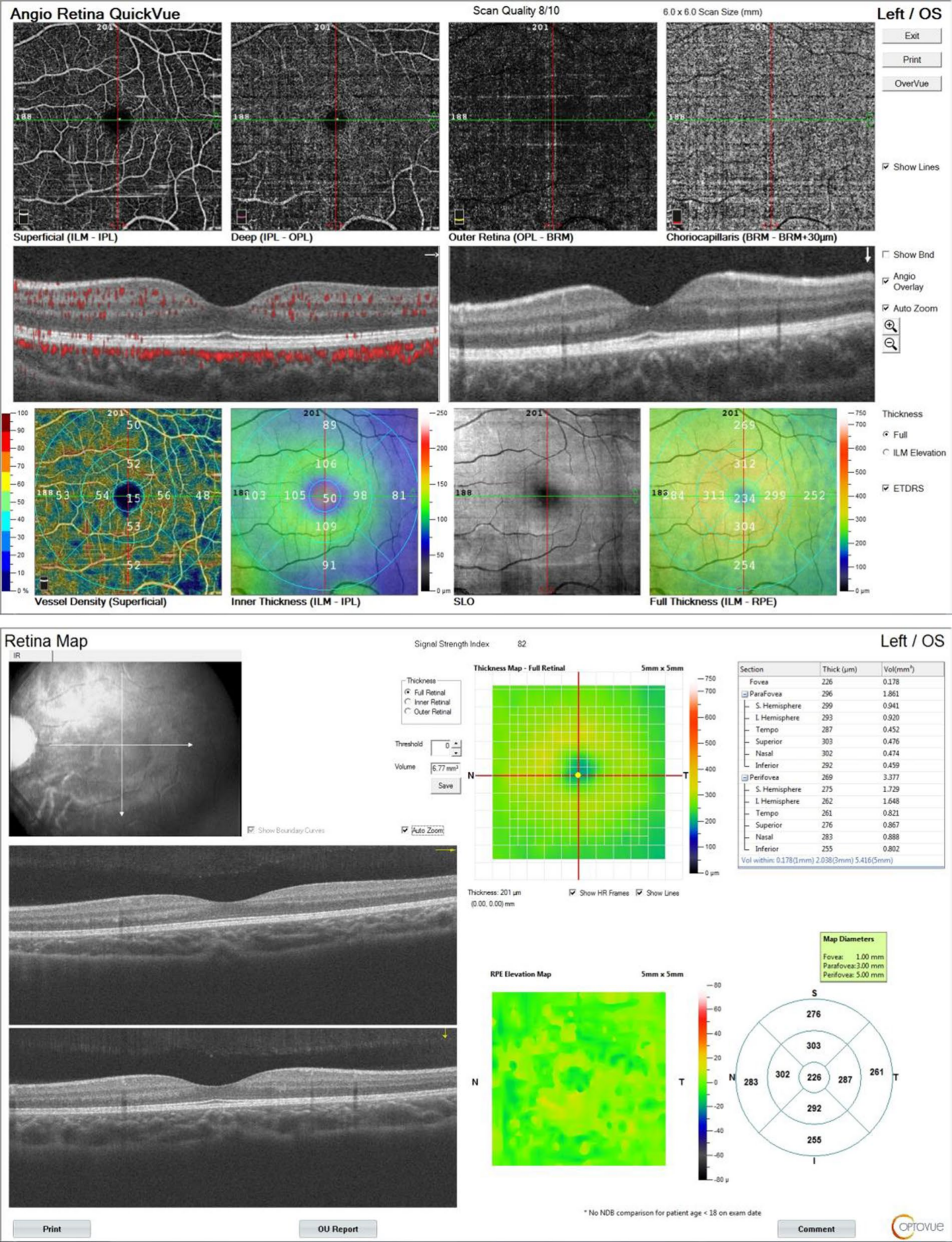
Images of vascular density and retinal thickness of macular area in healthy Chinese children. The AngioVue software automatically maps and calculates the percentages occupied by vessel density in superficial and deep capillary plexus

**Fig. 2:**
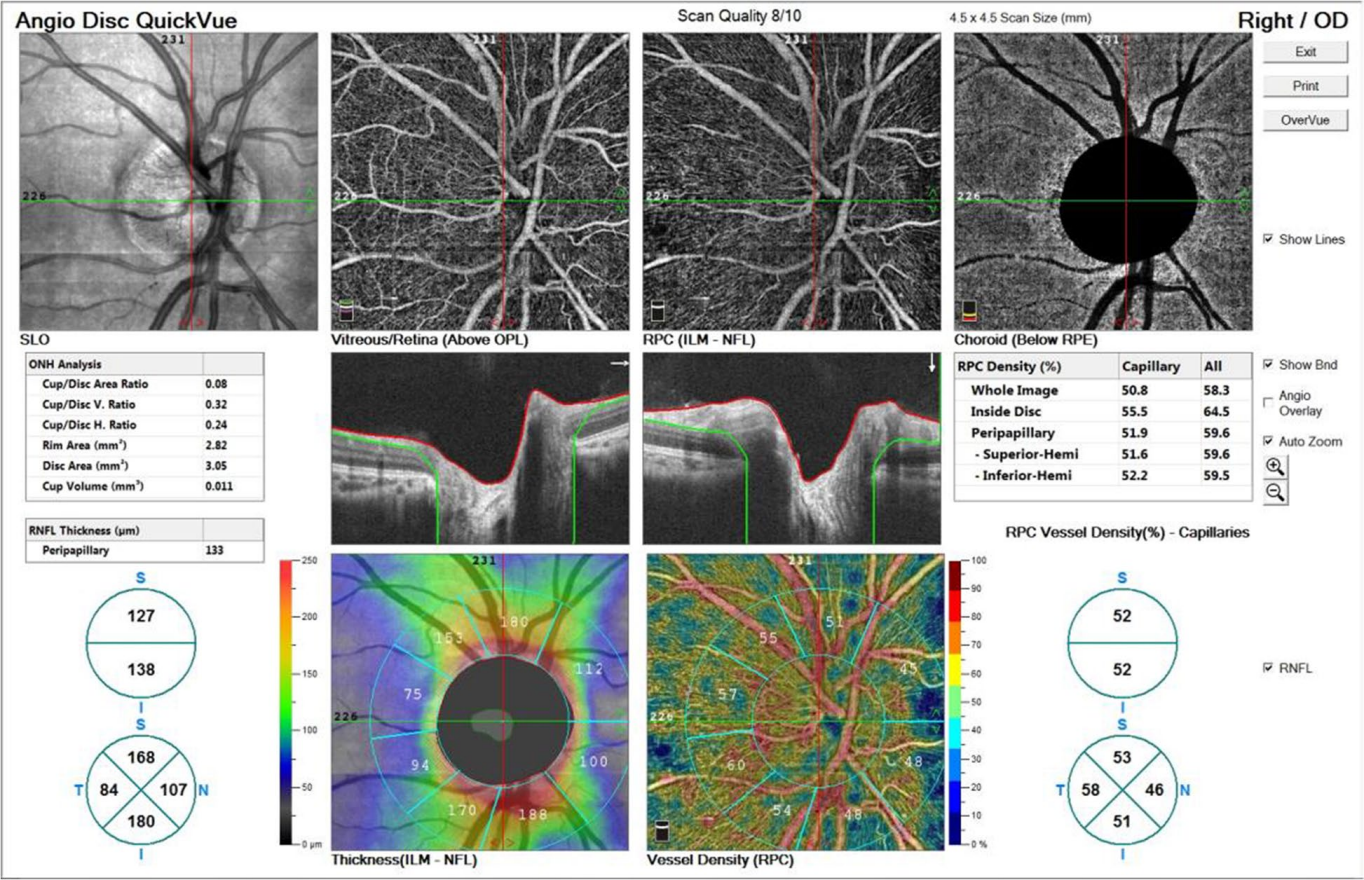
Images of vascular density and retinal thickness of optic disc in healthy Chinese children. The AngioVue software automatically maps and calculates the percentages occupied by vessel density in radial peripapillary capillary

A total of 343 eyes from 343 individuals participated in our study and underwent OCTA imaging (Figs. 1 and 2). Among these, 101 eyes were excluded due to poor scan quality (with poor cooperation or significant motion artifacts). The remaining 242 eyes of 242 individuals (116 males and 126 females, mean age ± SD 5.31 ± 0.73 years, range 4–6 years) were recruited for quantitative assessment. None of the images needed manual correction of the automated retinal layer segmentation. The ocular parameters and demographic characteristics of the participants are listed in Tables 1 and 2.

**Table 1:** Structural Characteristics of 4–6-year-old Chinese children

Variables	Values
IOP(mmHg)	15.59 ± 2.92
CCT(μm)	535.92 ± 28.87
AD(mm)	2.80 ± 0.25
LT(mm)	3.70 ± 0.20
AL(mm)	22.40 ± 0.73
Disc area(mm^2^)	2.24 ± 0.44
Cup area(mm^2^)	0.48 ± 0.29
Rim area(mm^2^)	1.87 ± 0.37

*IOP* Intraocular pressure, *CCT* Central corneal thickness, *AD* Anterior chamber depth, *LT* Lens thickness, *AL* Axial length

Data are mean ± standard deviation (SD)

**Table 2:** Demographics of preschool Chinese children stratified by age

Age (y)	4	5	6
IOP (mmHg)	15.42 ± 3.13	16.06 ± 2.84	15.30 ± 2.88
CCT (um)	532.28 ± 34.98	532.82 ± 25.95	539.62 ± 28.37
AD (mm)	2.79 ± 0.20	2.75 ± 0.27	2.84 ± 0.25
LT (mm)	3.70 ± 0.19	3.72 ± 0.18	3.69 ± 0.22
AL (mm)	22.29 ± 0.59	22.23 ± 0.76	22.51 ± 0.67
spherical equivalent (D)	2.11 ± 0.24	1.89 ± 0.36	1.71 ± 0.37
Scan quality	6.90 ± 1.07	7.32 ± 1.11	7.42 ± 1.11

*IOP* Intraocular pressure, *CCT* Central corneal thickness, *AD* Anterior depth, *LT* Lens thickness, *AL* Axial length

Data are mean ± standard deviation (SD)

We summarized the distributions of retinal thickness and vessel density in Tables 3 and 4. The mean SCP was 48.10 ± 2.92%, the mean DCP was 48.74 ± 6.51%, the fovea values were lowest (21.16 ± 6.65% and 33.15 ± 7.99%), the parafovea values were highest (49.94 ± 3.79% and 51.93 ± 6.10%), and the values decreased outward. The mean whole image capillary of the RPC was 47.17 ± 2.52%, and the mean RNFL thickness was 96.42 ± 10.97 μm. Both the RPC vessel density and RNFL thickness were highest in the inferotemporal region (52.41 ± 5.05% and 133.77 ± 17.65 μm), followed by 51.48 ± 4.63% and 127.48 ± 16.11 μm in the superotemporal region, respectively. The retinal vascular system of macula mainly spreads into two major plexuses: the SCP en face OCTA image was segmented with an inner boundary set at the ILM and an outer boundary at 10 μm below the inner plexiform layer (ILM to IPL-10), and the DCP en face OCTA image was segmented with an inner boundary 10 μm below the inner plexiform layer(IPL) and an outer boundary at 10 μm above the outer plexiform layer (OPL, IPL-10 to OPL + 10). Optic disc was recorded by whole image capillary, inside-disc capillary and RPC (a 1 mm-wide elliptical annulus extending from the optic disc boundary) in vasculature and RNFL in retinal thickness. The RPC segments extended from an inner boundary at the internal limiting membrane(ILM) to an outer boundary set at interface of nerve fiber layer (NFL).

**Table 3:** Descriptions of retinal thickness and vessel density in macula stratified by age and gender

		IRT (μm)			FRT (μm)			SCP(%)			DCP(%)		
		Male	Female	Total	Male	Female	Total	Male	Female	Total	Male	Female	Total
Average	4	98.95 ± 4.76	94.27 ± 5.85	96.31 ± 5.83	282.07 ± 11.21	279.46 ± 16.34	280.59 ± 14.22	49.32 ± 2.81	47.26 ± 2.15	48.16 ± 2.64	49.35 ± 5.33	46.25 ± 5.95	47.60 ± 5.83
	5	97.60 ± 6.24	99.53 ± 4.92	98.65 ± 5.61	282.52 ± 9.25	286.44 ± 9.54	284.66 ± 9.55	48.46 ± 2.51	47.21 ± 2.43	47.78 ± 2.53	47.45 ± 7.38	48.89 ± 6.39	48.23 ± 6.85
	6	100.39 ± 4.93	99.91 ± 5.75	100.15 ± 5.33	291.12 ± 9.84	287.06 ± 11.92	289.14 ± 11.04	49.00 ± 3.33	47.61 ± 3.09	48.33 ± 3.28	49.82 ± 6.00	49.19 ± 6.88	49.52 ± 6.42
	total	99.22 ± 5.50	98.78 ± 5.81	98.99 ± 5.66	286.83 ± 10.70	285.49 ± 12.22	286.13 ± 11.52	48.86 ± 2.99	47.39 ± 2.69	48.10 ± 2.92	48.94 ± 6.46	48.56 ± 6.58	48.74 ± 6.51
Center	4	47.75 ± 8.98	46.57 ± 5.88	47.09 ± 7.31	220.72 ± 19.83	222.63 ± 12.25	221.80 ± 15.79	19.82 ± 6.23	19.79 ± 5.37	19.81 ± 5.68	29.46 ± 8.70	32.17 ± 7.84	30.99 ± 8.23
	5	50.53 ± 9.54	47.17 ± 7.98	48.70 ± 8.83	230.36 ± 15.60	226.18 ± 15.96	228.08 ± 15.84	22.12 ± 6.04	20.59 ± 5.22	21.29 ± 5.63	35.29 ± 6.59	31.85 ± 6.82	33.42 ± 6.90
	6	52.36 ± 9.31	48.53 ± 8.64	50.50 ± 9.16	236.08 ± 20.92	226.92 ± 15.56	231.62 ± 18.99	22.98 ± 8.11	19.97 ± 6.77	21.51 ± 7.61	35.55 ± 7.94	31.69 ± 8.93	33.67 ± 8.62
	total	51.05 ± 9.40	47.67 ± 7.95	49.29 ± 8.82	231.86 ± 19.65	225.89 ± 15.16	228.75 ± 17.68	22.22 ± 7.22	20.18 ± 5.95	21.16 ± 6.65	34.57 ± 7.85	31.84 ± 7.93	33.15 ± 7.99
parafovea	4	105.27 ± 6.66	99.00 ± 4.97	101.73 ± 6.49	306.91 ± 13.62	300.07 ± 15.67	303.05 ± 15.02	52.59 ± 3.83	48.87 ± 2.95	50.49 ± 3.81	54.22 ± 4.48	49.52 ± 6.29	51.58 ± 5.99
	5	103.95 ± 10.96	103.55 ± 5.31	103.73 ± 8.31	307.23 ± 10.83	308.59 ± 10.79	307.97 ± 10.77	50.17 ± 3.24	49.32 ± 2.65	49.70 ± 2.95	50.03 ± 6.70	52.06 ± 5.83	51.14 ± 6.29
	6	106.67 ± 6.24	104.83 ± 5.07	105.78 ± 5.75	315.27 ± 11.00	309.71 ± 12.74	312.56 ± 12.16	50.41 ± 4.76	49.45 ± 3.83	49.94 ± 4.34	52.54 ± 7.94	52.78 ± 5.75	52.66 ± 5.96
	total	105.53 ± 8.25	103.33 ± 5.51	104.38 ± 7.03	311.27 ± 11.97	307.60 ± 12.99	309.36 ± 12.62	50.65 ± 4.21	49.29 ± 3.25	49.94 ± 3.79	51.92 ± 6.30	51.94 ± 5.95	51.93 ± 6.10
perifovea	4	98.98 ± 5.27	94.65 ± 6.87	96.54 ± 6.52	276.92 ± 11.95	275.44 ± 17.26	276.09 ± 15.01	50.19 ± 2.92	48.41 ± 2.37	49.19 ± 2.94	49.21 ± 5.72	45.97 ± 6.54	47.39 ± 6.33
	5	97.49 ± 5.51	100.29 ± 5.32	99.02 ± 5.55	277.08 ± 9.29	282.08 ± 9.78	279.81 ± 9.83	49.36 ± 2.54	47.89 ± 2.53	48.56 ± 2.62	47.08 ± 8.08	48.83 ± 6.73	48.04 ± 7.38
	6	100.34 ± 5.05	100.39 ± 6.80	100.36 ± 5.94	285.90 ± 9.99	282.52 ± 12.82	284.26 ± 11.53	49.80 ± 3.09	48.29 ± 3.09	49.07 ± 3.17	49.90 ± 6.63	49.20 ± 7.46	49.56 ± 7.02
	total	99.15 ± 5.36	99.35 ± 6.61	99.26 ± 6.03	281.54 ± 10.93	281.12 ± 12.85	281.32 ± 11.95	49.71 ± 2.88	48.16 ± 2.76	48.90 ± 2.91	48.83 ± 7.11	48.50 ± 7.08	48.66 ± 7.08

*IRT* Inner retinal thickness, *FRT* Full retinal thickness, *SCP* Superficial capillary plexus, *DCP* Deep capillary plexus;

Data are mean ± standard deviation (SD)

**Table 4:** Descriptions of retinal thickness and vessel density in optic disc stratified by age and gender

		RPC density (%)			RNFL thickness (μm)		
		Male	Female	Total	Male	Female	Total
Whole-	4	48.93 ± 2.47	46.68 ± 1.53	47.67 ± 2.27	99.69 ± 14.28	103.84 ± 12.17	102.03 ± 13.12
image	5	47.83 ± 2.61	45.91 ± 1.83	46.79 ± 2.40	103.55 ± 9.52	104.16 ± 11.42	103.88 ± 10.54
	6	47.93 ± 2.44	46.63 ± 2.72	47.29 ± 2.65	103.26 ± 6.52	105.08 ± 13.12	104.15 ± 10.27
	total	48.05 ± 2.51	46.37 ± 2.24	47.17 ± 2.52	102.84 ± 9.07	104.52 ± 12.25	103.53 ± 10.57
Inside-	4	49.21 ± 5.27	46.21 ± 5.01	47.52 ± 5.28			
disc	5	49.13 ± 4.14	47.49 ± 3.82	48.24 ± 4.03			
	6	47.31 ± 5.06	48.68 ± 3.84	47.98 ± 4.54			
	total	48.21 ± 4.84	47.80 ± 4.13	47.99 ± 4.48			
peripapillary	4	50.55 ± 3.39	47.88 ± 1.96	49.04 ± 2.96			
	5	48.75 ± 2.89	46.85 ± 2.39	47.71 ± 2.79			
	6	49.58 ± 3.15	47.83 ± 3.11	48.73 ± 3.24			
	total	49.44 ± 3.13	47.46 ± 2.70	48.41 ± 3.07			

Data are mean ± standard deviation (SD)

**Fig. 3:**
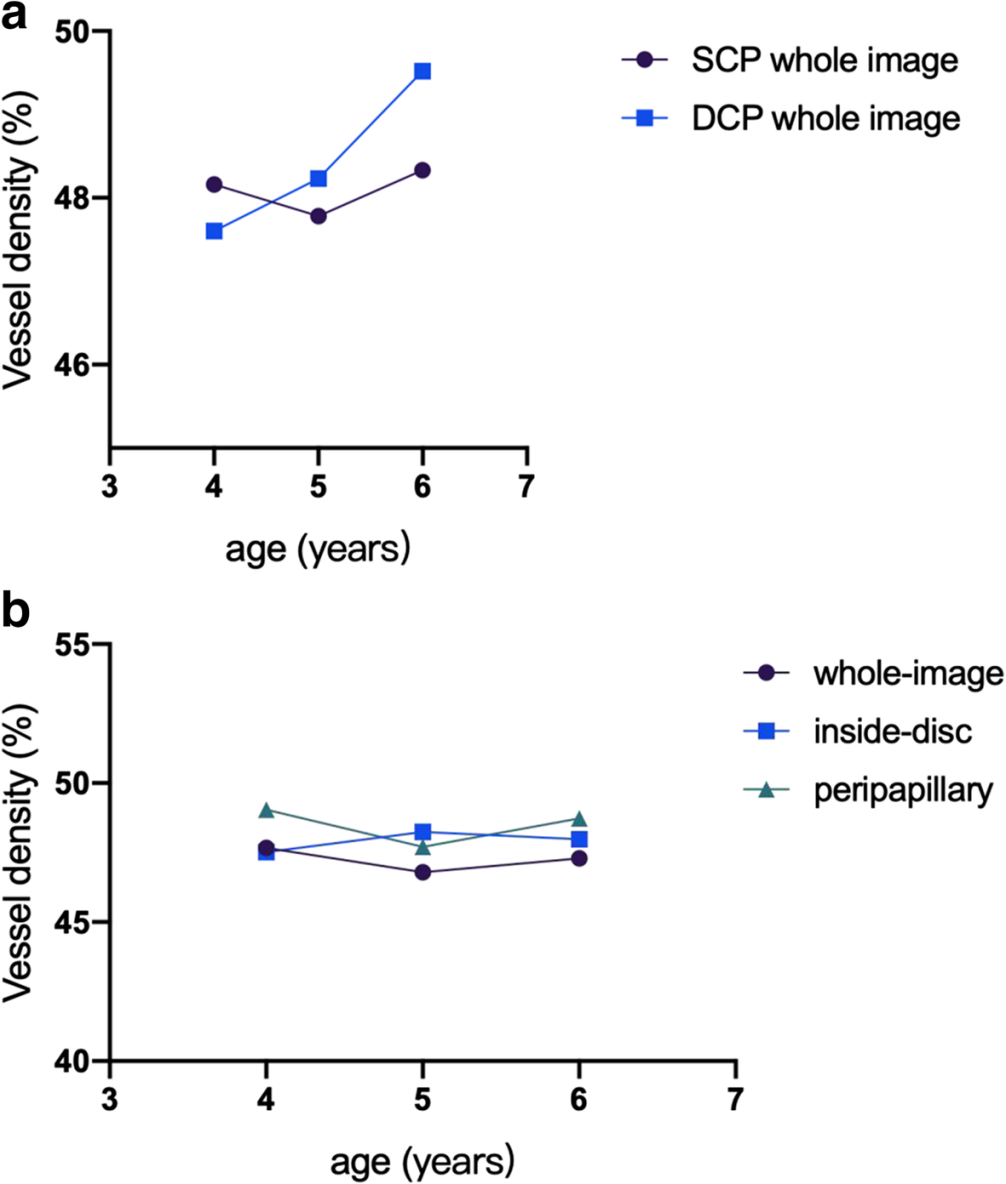
Distribution of the retinal vasculature stratified by age

In the evaluation of age-related parameters, a slightly significant correlation between the age of participants and DCP (r = 0.117, *P* = 0.068, Fig. 3) was found but not between the FAZ area, SCP, and peripapillary RPC (*P* > 0.05). Values of retinal vessel density and thickness were similar in the right and left eyes (P > 0.05) but not in the male and female participants. The peripapillary RPC, SCP, and DCP values were significantly higher in males than in females (*P* < 0.001). In the univariate linear regression analysis, the rim area was positively correlated with the peripapillary RPC (standardized coefficients β = 0.195, *P* = 0.002). Axial length was positively correlated with the peripapillary RPC, SCP, and DCP (standardized coefficients β = 0.618, *P* < 0.001; β = 0.591, *P* < 0.001; β = 0.232, *P* < 0.001). Other correlations in the multivariate linear regression analysis are listed in Table 5.

**Table 5:** Multivariate linear regression Analysis of the retinal vasculature

	Parameters	Standardized Coefficients β	*P* Value
Associations with	CCT	0.007	0.908
mean DCP	LT	−0.001	0.994
	AD	−0.010	0.914
	AL	0.225	0.002
	IOP	−0.051	0.440
Associations with	CCT	0.049	0.343
mean SCP	LT	−0.024	0.711
	AD	0.136	0.057
	AL	0.516	< 0.001
	IOP	−0.179	0.001
Associations with	CCT	− 0.016	0.752
peripapillary	LT	0.060	0.360
capillary	AD	−0.041	0.560
	AL	0.652	< 0.001
	IOP	−0.049	0.350

*IOP* Intraocular pressure, *CCT* Central corneal thickness, *AD* Anterior chamber depth, *LT* Lens thickness, *AL* Axial length

Data are mean ± standard deviation (SD)

**Fig. 4:**
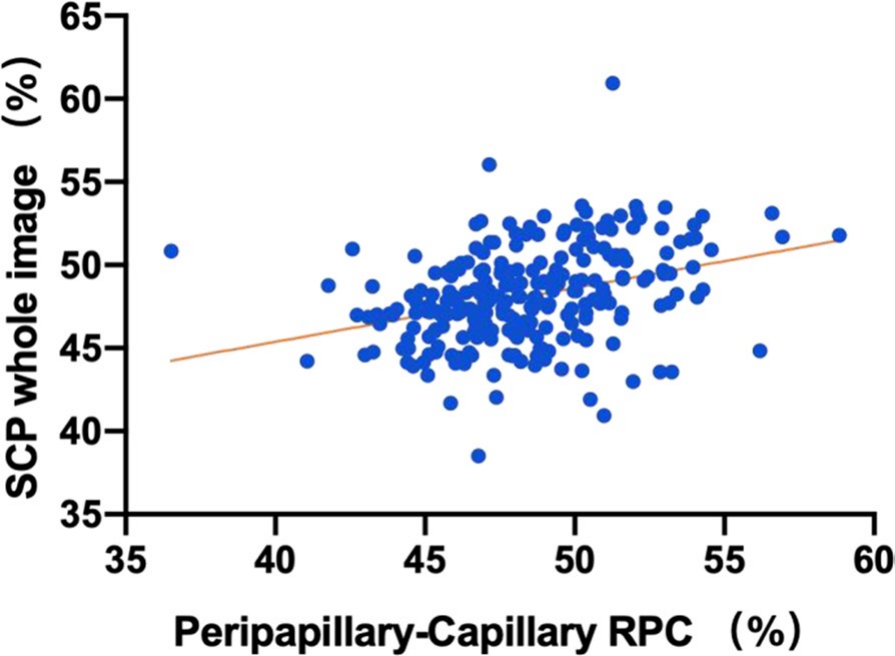
Correlation between the mean SCP and peripapillary RPC

**Fig. 5:**
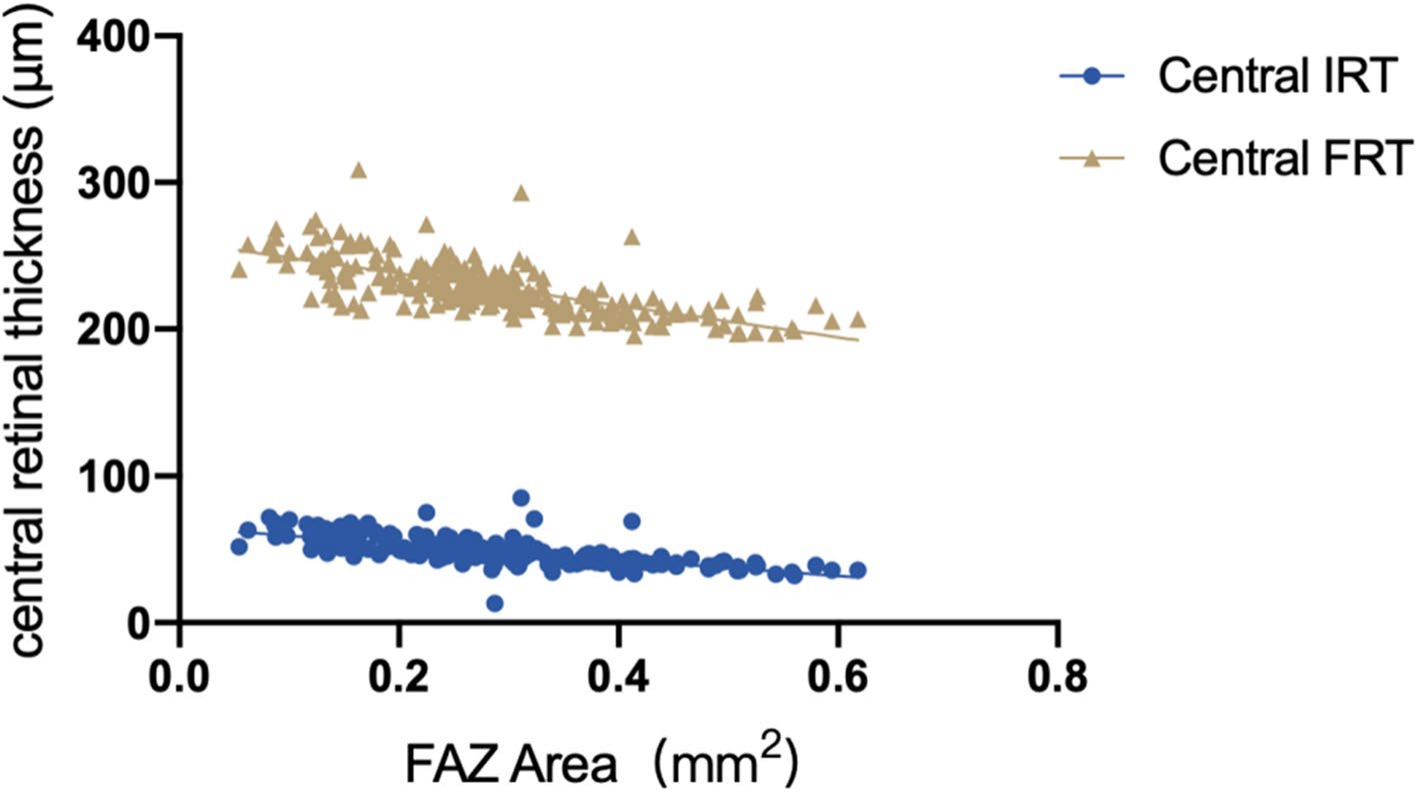
Correlation between the FAZ area and central IRT and FRT

**Fig. 6:**
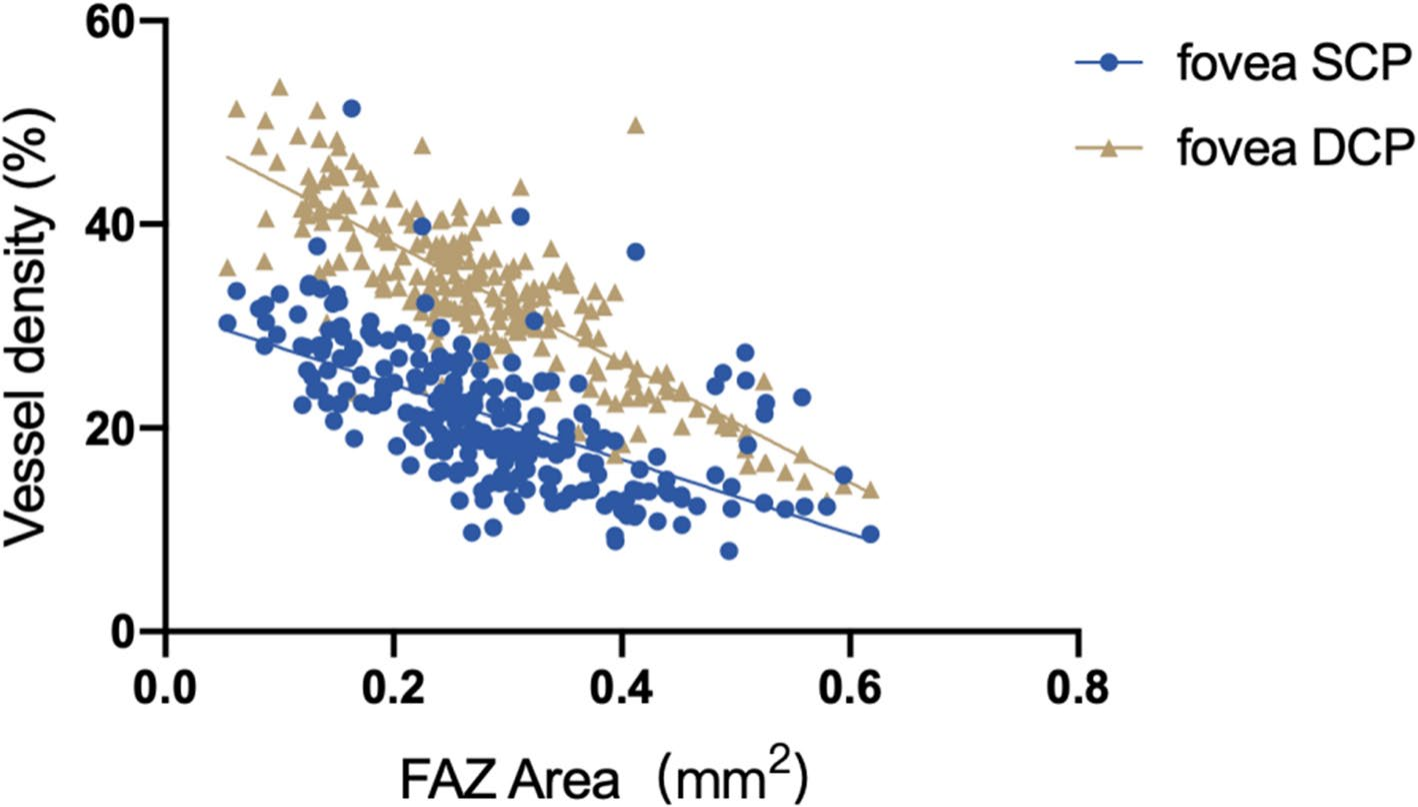
Correlation between the FAZ area and foveal SCP and DCP

In the Spearman correlation analysis (Table 6), the mean SCP was correlated with peripapillary capillary RPC (r = 0.380, *P* < 0.001, Fig. 4). It was not significantly correlated with the mean DCP, mean SCP, or retinal thickness (*P* > 0.05). The FAZ area was similar in the left and right eyes, as well as in the male and female eyes (*P* > 0.05). It was inversely correlated with the central inner retinal thickness (r = − 0.793 *P* < 0.001, Fig. 5) and full retinal thickness (r = − 0.737 *P* < 0.001, Fig. 5) and significantly negatively correlated with the foveal SCP (r = − 0.683 *P* < 0.001, Fig. 6) and DCP (r = − 0.784 *P* < 0.001, Fig. 6).

**Table 6:** Correlation between retinal thickness and vasculature

		SCP whole image (%)	DCP whole image (%)	Peripapillary capillary RPC (%)	FAZ area (mm^2^)	RNFL (μm)	IRT (μm)	FRT (μm)
SCP whole	correlation coefficient	1.000	.322^**^	.380^**^	−.030	−0.16	.088	−.005
image (%)	Sig. (two-tails)	.	.000	.000	.642	.807	.174	.940
DCP whole	correlation coefficient	.322^**^	1.000	.078	−.008	.015	−.077	−.075
image (%)	Sig. (two-tails)	.000	.	.226	.896	.819	.231	.245
Peripapillary	correlation coefficient	.380^**^	.078	1.000	−.113	−.051	−.063	−.118
capillary RPC (%)	Sig. (two-tails)	.000	.226	.	.080	.425	.327	.066
FAZ area	correlation coefficient	−.030	−.008	−.113	1.000	.060	−.003	−.023
(mm^2^)	Sig. (two-tails)	.642	.896	.080	.	.350	.960	.717
RNFL (μm)	correlation coefficient	−.016	.015	−.051	.060	1.000	.000	−.027
	Sig. (two-tails)	.807	.819	.425	.350	.	.997	.671
IRT (μm)	correlation coefficient	.088	−.077	−.063	−.003	.000	1.000	.685^**^
	Sig. (two-tails)	.174	.231	.327	.960	.997	.	.000
FRT (μm)	correlation coefficient	−.005	−.075	−.118	−.023	−.027	.685^**^	1.000
	Sig. (two-tails)	.940	.245	.066	.717	.671	.000	.

*IRT* Inner retinal thickness, *FRT* Full retinal thickness, *SCP* Superficial capillary plexus, *DCP* Deep capillary plexus

Data are mean ± standard deviation (SD)

## Discussion

Various notable studies have investigated retinal thickness and vasculature in healthy adolescents [25, 27–29] and adults [26, 32–39]. This study provides new information regarding young children. We found a significant difference between male and female eyes in retinal vasculature (SCP, DCP, and RPC), with male vessel densities higher than female vessel densities. This result might be due to the serum insulin-like growth factor (IGF-1) increase in males during 4–6 years old [37]. In contrast, we did not observe that age was significantly correlated with vasculature in the macular and disc areas, except that the mean DCP was slightly increased with aging; however, variable relationships were assessed in other research. Hsu et al. [25] analyzed 9-week- to 17-year-old infancies and adolescents and showed a quadratic relationship in which macular perfusion was enhanced by aging until almost 10 to 15 years old, after which it started to decrease. Qi et al. [33] investigated 1631 Chinese subjects 18–90 years old and found that SCP and DCP increased with age until 60 (47.53%) and 50 (56.73%) years old, respectively, and then decreased. Compared to SCP and DCP in the research of Zhang et al. [27] and Coscas et al. [34], our results seemed relatively low. We inferred that the macular vasculature increased with aging in an early life stage and then decreased after the peak. Despite the fact that significant results were obtained above, we could not determine the process’s definite inflection point. In the evaluation of the vasculature in the disc area, other studies performed a correlation between peripapillary RPC and age. Zhang et al. [29] noticed a stable trend in children aged 5–18 years; Jo et al. [26] recruited healthy adults 18–83 years old and found an inverse association between age and peripapillary capillary density in almost all sectors. The relationships seemed comparable to the findings in the macula (vascular density increased with aging and then decreased with eye age). As a result, the developmental maturity of retinal vasculature in different regions is accomplished at almost the same periods, which might be due to elevation of certain growth-related hormones in preschool children [40].

Regarding FAZ, its area ranges from 0.25 mm^2^ to 0.32 mm^2^ in studies that recruited more than 50 healthy subjects [27, 28, 38, 41–45], which is comparable to the current study (0.30 ± 0.13 mm^2^). There seemed to be no significant relationships between age and the FAZ area in several previous studies [25, 42, 45, 46] and this one. Some researchers such as Yu et al. [38] and Pujari et al. [47] found a positive correlation across the entire life span. Consequently, according to the results from these studies [25, 27, 28, 41–47], the FAZ area does not decrease with age. It appears to be basically mature before 4–6 years old and reaches a steady state or slightly decreases before complete development. Afterward, it begins to increase as eyes age.

In addition, it was recognized that the DCP was higher than the SCP in almost all macular regions in previous studies, and our results are in line with this finding [14, 27]. Morphological features lead to vascular discrepancies in which the DCP consists of subtle capillary vortices and branches converging radially toward the epicenter, but linear structure capillaries form the SCP [34, 48]. Therefore, in contrast with SCP, DCP tends to suffer a significant influence under relatively low vasculature. Kim et al. [49] found that DCP decreased preceding SCP in diabetic eyes, and circulatory disturbance occurred before structural damage. Hsu et al. [20] and Koullsis et al. [19] also revealed that DCP decreased in FEVR eyes, and macular vasculature changes could reflect peripheral vascular changes using OCTA. As a result, when the deviation of vascular values is observed, especially in DCP, it should be noted whether it is due to the abnormality in growth or damage in retinal structure.

With regard to retinal thickness and vessel density correlations and distributions, many studies have analyzed their correlations in adolescents and adults [27, 46, 50–55]. Zhang Z et al. [27] found that central macular thickness was positively correlated with foveal SCP and DCP. She et al. [54] suggested that the peripapillary RPC showed a positive correlation with RNFL thickness. According to the studies above, we expected to find similar relationships in our results. However, there was no significant correlation between the macular vasculature and retinal thickness or between the RPC density and RNFL thickness (*P* > 0.05). Notably, the retinal vasculature is the highest metabolic network in the human body, and angioactive (angiotensin, NO), local (IOP, AL), and systemic (blood pressure, diabetes) factors regulate the circulation [38]. This might be due to the discrepancy in the factors above among children, adolescents, and adults, leading to childhood insignificance. In addition, Fujiwara et al. [46] observed that the FAZ area was inversely correlated with central retinal thickness in the inner retinal thickness and full retinal thickness and foveal vessel density in both the SCP and DCP. These findings are comparable to ours with the concept that central retinal vasculature supplies the inner retinal thickness. The interaction between the inner retinal thickness and full retinal thickness and the correlation between retinal vessel density and retinal thickness might explain the current findings. Rezar et al. [21] recently reported that the FAZ area in ROP eyes is relatively narrowed, as well as the FAZ perimeters. Araki et al. [56] found that amblyopic eyes showed a smaller FAZ area but no significant difference in the macular vasculature even after magnification error correction. Many other studies [57] in children also did not find a significant correlation in macular vasculature, but they believed that the localized defects may be specific. However, Demirayak et al. [58] showed decreased whole and parafoveal DCP and a smaller FAZ area in adult amblyopia. As FAZ area and macular vessel density were associated with poor visual acuity, we suspected that FAZ area reduction and macular vasculature defects were the initiating factors or the results of amblyopia. In the latest study, Wong et al. [59] found that the FAZ circularity and vessel density index could be sensitive markers to assess amblyopia in 30 participants with amblyopia. The large sample and multi-center studies may have more scientific guiding effect on the change of FAZ area in children with amblyopia.

Thus, an understanding of the FAZ area in children might be required in future studies. The FAZ area parameter might be used as an objective marker in the diagnosis and monitoring of abnormalities in children.

We further observed that the mean SCP was positively correlated with peripapillary RPC. Yarmohammadi et al. [60] studied the vessel densities in glaucoma and found that the vessel densities in both macular and optic discs showed a reduction, especially in the affected hemiretinae with hemifield loss. Another study showed that the peripapillary RPC was significantly higher in well-controlled primary angle-closure glaucoma (PACG) than in poorly controlled PACG [61]. We further found a positive correlation between the rim area and peripapillary RPC in the univariate and multivariate regression analyses. Iwase et al. revealed the small rim area as a new risk factor for glaucoma [62], which might be a significant sign for the development and progression of glaucoma if confirmed in the future. However, these findings were found in adults and further researches are needed on whether they could apply to children. Because retinal thickness is still growing and developing, the reduction in the RNFL may not be significant, but vascular changes, such as SCP and peripapillary RPC, may occur first. In addition, if the rim area—the high-risk factor for glaucoma—is reduced, close follow-up may be required to detect pathological changes early. Thus, the correlations must be considered when assessing and interpreting the vascular alterations in children’s possible pathologic processes. Taken together, these findings warrant further investigation to acquire definite results and correlations for diagnostic applications in retinopathy and developmental stagnation of preschool children.

A longitudinal study described the emmetropization process: the rapid early progression pattern was followed by slower growth at older ages from 3 months to 6.5 years old, which led to AL elongation, AD deepening, and LT thinning [63]. We found a positive correlation between the AL and retinal vasculature (SCP, DCP, and RPC). Although such a relationship may occur after the magnification error is corrected, it may also be a real correlation. It shows that the anterior and posterior segments of the children’s eyes develop simultaneously at this stage, although the correlation with age is not significant. However, a negative relation between the AL and retinal vasculature was found using a computer-based program to eliminate the magnification error in OCTA images [64–66]. This discrepancy might derive from the different machines, methods, and units of the retinal vessel. As these studies’ small sample size could lead to result error, future studies with large samples and multiple centers are required.

Children are liable to become refractive errors, especially myopia, and experience rapid myopia progression because of sensitivity to environmental factors (e.g., heavy homework, competitive lifestyles, and few outdoor activities) in Asian countries [67, 68] during the emmetropization process. When an abnormality occurs, the parameter values in the anterior segment and the posterior segment will deviate from the normal value. Our normal parameter values may provide a reference for monitoring refractive development in children during this period.

Despite the strengths of our study, limitations should be noted. First, OCTA technology has inherent disadvantages of motion artifacts, superficial vascular projection, and limited scanning range. Second, the refractive development of healthy children aged 4–6 years old is in the process of emmetropization; thus, our study was limited to hyperopia, causing a lack of the relevant retinal parameters detected in myopia. Last but not least, the narrow racial and age band limited the results’ application, thus other populations need further studies.

## Conclusions

In conclusion, our study demonstrated macular and optic disc reference values in 4- to 6-year-old healthy preschool children in China after magnification error was corrected by Littman and the modified Bennett formula. Overall, the density of DCP was higher than that of SCP, and the RPC density performed highest in the inferotemporal. There seemed to be an inverse correlation between the FAZ area and foveal SCP or DCP. Simultaneously, age seemed to be slightly positively related to DCP, but other vascular parameters, such as SCP and RPC vessel density, were not age-related. The current data and these relevant correlations should be considered in longitudinal OCTA studies and clinical applications.

## Data Availability

Data used by this study are available from the corresponding author upon reasonable request.
